# The effects of a real-life lifestyle program on physical activity and objective and subjective sleep in adults aged 55+ years

**DOI:** 10.1186/s12889-022-12780-2

**Published:** 2022-02-19

**Authors:** J. Vanderlinden, F. Boen, S. Van Puyenbroeck, J. G. Z. van Uffelen

**Affiliations:** 1 Department of Movement Sciences, Physical Activity, Sports and Health Research Group, KU Leuven, University of Leuven, Tervuursevest 101 box 1500, B-3000 Leuven, Belgium; 2 Department of Health, Odisee University for Applied Sciences, B-1000 Brussels, Belgium

**Keywords:** Sleep, Accelerometer, PSQI, Older adults, Elderly, Health promotion, Lifestyle, Intervention, Ageing

## Abstract

**Study objectives:**

Age related changes in sleep result in an increasing prevalence of poor sleep in mid-aged and older adults. Although physical activity has shown to benefit sleep in studies in controlled settings, this has not yet been examined in a real-life lifestyle program. The aims of this study were to: 1) examine the effects of a lifestyle program on moderate-to-vigorous physical activity and objective and subjective sleep in adults aged 55+ years; and 2) examine if the effects differed between good and poor sleepers.

**Methods:**

This controlled pretest-posttest trial examined the effects of the 12-week group-based real-life lifestyle program ‘Lekker Actief’ on moderate-to-vigorous physical activity (measured using accelerometers) and sleep (measured using accelerometers and the Pittsburgh Sleep quality Index, PSQI). The main component of the program was a 12-week progressive walking program, complemented by an optional muscle strengthening program and one educational session on healthy nutrition. Of the 451 participants who were tested pre-intervention, 357 participants completed the posttest assessment (200 in the intervention group and 157 in the control group). Effects on moderate-to-vigorous physical activity and on objective sleep (sleep efficiency, total sleep time, wake time after sleep onset (WASO) and number of awakenings) as well as subjective sleep (sleep quality) were examined in crude and in adjusted multiple regression models. An interaction term between program (control versus intervention) and sleep category (good and poor) was included in all models.

**Results:**

Moderate-to-vigorous physical activity levels significantly increased in the intervention group compared with the control group (43,02 min per day; 95%CI: 12.83–73.22; fully adjusted model). The interaction terms revealed no differences between good and poor sleepers regarding the effect of the intervention on moderate-to-vigorous physical activity. There were no significant effects on sleep, except for good sleepers who showed an increase in number of awakenings/night by 1.44 (CI 95% 0.49; 2.24).

**Conclusions:**

Although this program was effective in increasing physical activity, it did not improve sleep. Lifestyle programs should be promoted to increase physical activity, but more is needed to improve sleep as well.

This trial was registered at ClinicalTrials.gov (Trial registration NCT03576209).

## Background

Healthy ageing has been described by WHO in 2015 as ‘the process of developing and maintaining the functional ability that enables wellbeing in older age’ [1]. Physical activity (PA) is an important determinant of healthy ageing as it has been positively associated with several health outcomes in adults, such as a decreased risk for all-cause mortality, diabetes type 2, cardiovascular disease, cancer and hypertension [2, 3]. Moreover, regular PA has shown to improve aspects of physical and mental health that are particularly important for older adults, such as balance, cognitive function, functional ability, sleep and the immune system [4, 5].

PA has been defined as any bodily movement produced by skeletal muscles that results in energy expenditure [6]. Despite the established positive associations between regular PA and health outcomes, the proportion of people meeting the World Health Organization (WHO) recommendations of at least 150 min of moderate intensity physical activity (MPA), or 75 min vigorous intensity physical activity (VPA) per week decreases with age [7].

Besides PA, sleep is also a crucial factor in terms of healthy ageing [5, 8, 9]. Good sleep has been shown to improve cognitive functioning, mental well-being, ability to perform activities of daily living and self-reported health status and to reduce the risk of falling and hospitalization [8, 10]. As of the age of 60 years, sleep quality and quantity tend to decrease [1, 11], which in turn results in a prevalence of sleep problems of 50% in older adults [11, 12]. Besides other non-pharmaceutical treatment options for sleep problems in older adults (such as hygiene education, cognitive behavioural therapy and relaxation [5, 13, 14], also PA has been shown to benefit sleep outcomes in older adults, such as wake time after sleep onset (WASO), sleep quality, sleep latency, sleep efficiency and sleep disturbances [5, 15, 16]. The biological mechanisms that underlie the positive effects of regular PA on sleep include relaxation which helps to prepare body and mind for a good night sleep; and an increased energy expenditure that increases sleep pressure and in turn creates physiological tiredness [16–18]. Being physically active outdoors may also increase exposure to bright day light, which helps to reset the circadian rhythm and can in turn enhance sleep [19, 20].

Current evidence from randomized clinical trials and pragmatic clinical trials indicates that participation in controlled physical activity programs has a beneficial effect on sleep in older adults [5]. However, to our knowledge, these findings have not yet been replicated in a real-life lifestyle program including a physical activity component. As the overarching aim of research is to implement interventions that have been proven effective in RCTs, the aim of this study was to examine the effect of a real-life lifestyle program, offered by a large senior citizen organisation, on physical activity and sleep in older adults.

It should also be noted that both physical activity and sleep outcomes in these studies were mainly assessed by subjective measuring methods only, namely surveys. Although correlations between subjective and objective measures of sleep have shown mixed results in literature [21–23] both types of measurement represent different aspects of sleep and can provide complementary information in understanding sleep [24–26]. Moreover, most of these studies did not separately analyse the explicit effects of a physical activity program on sleep in either good or poor sleepers in those programs [27, 28]. Finally, the vast majority of the current studies examining the effects of physical activity programs on sleep were studies in a controlled or experimental setting with rather small samples of older adults (between *n* = 13 and *n* = 128) [5], limiting the generalisability of these results to PA programs in real-life settings.

Given the growing life expectancy and the increased prevalence of poor sleep and sleep problems at higher age; as well as the emerging importance of sleep in terms of healthy ageing, there is a need for evidence on the effectiveness of interventions to promote PA and sleep in a real-life setting. Consequently, examining effects of real-life PA programs in larger samples of older adults, using both objective and subjective measuring methods to assess PA and sleep outcomes, are critical to fill this gap in the current knowledge.

In this study, we will examine the effects of a 12-week group-based lifestyle program in a real-life setting (‘Lekker Actief’). This lifestyle program was provided by a socio-cultural organisation OKRA SPORT+ to adults aged 55 + years in Flanders, Belgium.

The aim of this study were to examine the effects of the lifestyle program ‘Lekker Actief’ on device-measured MVPA and objective and subjective sleep in adults aged 55+ years; and to examine if the effects differed between good and poor sleepers.

## Methods

### Design

This study used a controlled pretest-posttest design. Data from participants in the intervention and control groups were collected at baseline (T0, pretest) and posttest (T1) with an in between time interval of 12 weeks. The participants in the intervention group participated in the lifestyle program ‘Lekker Actief’ for a period of 12 weeks. The participants in the control group did not participate in the program and were asked to continue their usual activities. This trial was registered at ClinicalTrials.gov. (Trial registration NCT03576209).

### Study sample

Data were collected from community-dwelling older adults (aged ≥55 years) from July 2018 to July 2019. All participants who were enrolled in this study belonged to regional meeting points, which existed before the start of the study, of a socio-cultural organization for older adults in Flanders, Belgium (OKRA SPORT+). This organization provides weekly meetings for the older adults in meeting points in local areas in Flanders. Participants for this study were recruited using convenience sampling at the meeting point level. Older adults belonging to OKRA SPORT+ meeting points that offered ‘Lekker Actief’ were invited to participate in the intervention group of this study. Older adults belonging to OKRA SPORT+ meeting points that did not participate in ‘Lekker Actief’ were invited to participate in the study as the control group. For this study, the meeting points in the intervention and control groups were then matched based upon their location in the different regions in Flanders. This was done to ensure that participants from both groups were equally divided over Flanders. All older adults were given information on the study during one of the weekly meetings in their own meeting point. Subsequently, older adults were recruited in their meeting points. Inclusion criteria were being 55 years or older and being able and willing to participate in this study. Exclusion criteria for participation in the Lekker Actief program and this study were not being able to attend the OKRA SPORT+ meetings due to limited physical mobility. The ethics committee of UZ Leuven granted approval for this study (Ref. no: S61581). All participants received study information prior to the study and granted their written informed consent. All methods were carried out in accordance with relevant guidelines and regulations under ethical approval and consent to participate.

### Lifestyle program ‘Lekker Actief’

‘Lekker Actief’ was developed and delivered in 2018–2019 by OKRA SPORT+ to adults aged 55+ years. The primary aim of ‘Lekker Actief’ was to help adults aged 55+ years meet the recommended minimum MVPA-levels [29, 30]. This 12-week lifestyle program consisted out of a main walking component (i.e. a group-based peer-led structured walking program), and two minor components (i.e. a group-based peer-led muscle strengthening program and one session on healthy nutrition). There were no specific components aimed at improving sleep.

“This ‘Lekker Actief’ program was designed based on the assumptions of self-determination theory (Ryan & Deci, 2000). This theory assumes that sustainable behavioural change can only be obtained if three basic and universal human needs are supported: the need of autonomy, the need for competence and the need for relatedness. The ‘Lekker Actief’ program was developed to facilitate satisfaction with each of these three basic needs. For example, participants had autonomy in when to complete their individualized walking schedule. The program also facilitated feelings of competence by tailoring the program according to participants’ initial walking ability and fitness and by structuring the build-up according to the principles of training progression. Finally, the program supported the need of relatedness by offering weekly group walks and meetings within the trusted social environment of the local community, supervised by a well-known peer leader.” [31].

#### Walking program

The main component of this lifestyle program was the 12-week structured progressive walking program that was offered at least once weekly with the aim to increase the participants’ MVPA levels. All meeting points were offered a guideline with separate walks each containing a certain number of aerobic steps. Every week, the number of prescribed steps per week was progressively increased over a 12-week period of time. All participants in the intervention group followed this weekly walking schedule based on their initial walking fitness as determined by a self-guided six-minute walking test [32, 33] prior to the start of the program.

#### Muscle strengthening program

The secondary component of this lifestyle program involved the muscle strengthening exercises. All meeting points were offered the opportunity to provide peer-led group-based sessions with muscle strengthening exercises once a week. Meeting points that offered these exercises, encouraged the participants to follow the strengthening session in group. The participants were also offered a home-based exercise manual to continue these exercises at home. The muscle strengthening exercise program was not as structured as the walking program considering meeting points were left free in their choice if and how to organise the muscle strengthening program.

#### Healthy nutrition

The third and minor component in this lifestyle program consisted out of one two-hour professional-led educational session on healthy nutrition. In addition, all participants received information on healthy nutrition at the beginning of the program.

### Measurement

Data were collected at baseline (T0) and at posttest after 12-weeks (T1) using self-administered questionnaires (demographics, Pittsburgh Sleep Quality Index (PSQI)) and accelerometers (MVPA, sleep efficiency, total sleep time, WASO and number of awakenings).

#### Demographics

Demographic variables (age, gender, education, living situation and professional status) as well as general health related variables (smoking, use of alcohol, caffeine or screen time before bedtime, use of sleep medication and presence of chronic conditions) were collected with a self-reported questionnaire. The defined categories for education were in line with the International Standard Classification of Education (ISCED) 2011 [34].

#### Device-measured MVPA levels

MVPA levels were measured through accelerometry (Actigraph type wGT3X-BT, Actigraphcorp, Pensacola, FL). The details of accelerometry measurement are explained under ‘Actigraphy’.

#### Subjective sleep quality

Subjective sleep quality was assessed with the PSQI questionnaire, a frequently used 19-item self-reported questionnaire in older adults [35]. The PSQI determines subjective sleep quality over the last month and contains seven subscales: sleep quality, sleep latency, sleep duration, habitual sleep efficiency, sleep disturbances, use of sleep medication, and daytime dysfunction. According to the PSQI scoring protocol, each subscale provides a sub score, leading to a total PSQI score that ranges from 0 to 21 points, with higher scores indicating worse sleep quality. A cut-off threshold of > 5 points indicates poor-quality sleep. The PSQI is easy to complete for older adults, and provides highly reliable and valid measures of sleep quality [35].

#### Objective sleep outcomes

Objective sleep outcomes (sleep efficiency, total sleep time, WASO and number of awakenings) were measured through accelerometry (Actigraph type wGT3X-BT, Actigraphcorp, Pensacola, FL).

### Actigraphy

The Actigraph wGT3X-BT is an accelerometer that records physical activity associated with daily activity and sleep [36]. All participants were asked to wear the Actigraph device on their non-dominant wrist for six consecutive days, of which two weekend days, and five nights. Previous work showed that Actigraphy sleep measurement during a minimum of five nights results in valid sleep measurement [37]. The coefficients of validity of actigraphy are higher compared with medical tests and psychological tests for sleep [38]. The Actigraph wGT3X-BT has been used in numerous studies to measure MVPA and sleep in older adults [21–23, 39, 40]. The Actigraph accelerometer is considered a reliable tool for measuring overall PA level and intensity-specific PA in adults under free-living conditions [24].

In line with previous research, we only analysed data of participants with a minimum wear time of four wear days of at least 10 h of waking wear time data [39, 41, 42]. Accelerometer data were processed using well established validated algorithms available in Actilife software package (Actilife, v6.13.4), namely the Choi algorithm for wear time validation [39] and the Cole-Kripke algorithm for sleep-wake identification [41]. The following tri-axial vector magnitude (VM3) cut-points for wrist-worn actigraphy in older adults were used to identify MVPA (≥3268 CPM) [40].

### Good and poor sleepers

Participants were categorized as good (*n* = 168) or poor sleepers (*n* = 99) based on their pretest PSQI an objectively measured sleep data. This was done using previously established cut-off points of either sleep quality or sleep quantity characteristics [35, 43]: participants were categorized a good sleepers if they had PSQI score ≤ 5 points, which is considered as good sleep quality or a sleep efficiency ≥85%, which is considered as efficient sleep [35, 43]. Participants were categorized as poor sleepers if they had a PSQI score > 5 points or a sleep efficiency < 85%.

### Statistical analyses

Baseline differences between the intervention and the control groups in demographics, MVPA levels and sleep characteristics were examined using T-tests (continuous variables) and Chi-square tests (categorical variables). Linear regression models were used to examine the effects of the lifestyle program ‘Lekker Actief’ on MVPA, objective sleep outcomes (sleep efficiency, total sleep time, WASO and number of awakenings) and the subjective sleep outcome (total PSQI score for sleep quality). Three multiple regression models were constructed for each outcome as follows: (1) a crude model including condition and baseline value of the outcome measure, (2) a partially adjusted model adjusted for the demographics (i.e., crude model adjusted for age, gender, education, living situation and professional status), and (3) a fully adjusted model (partially adjusted model adjusted for demographics and covariates that have been shown to impact sleep in previous research, namely smoking [44], alcohol intake [45], caffeine [46], screen time before bedtime [47], the use of sleep medication [48], and the presence of chronic conditions [49]. To test whether the intervention effect differed between good and poor sleepers, an interaction term program (intervention versus control) by sleeping status (good versus poor) was added to all models. For those models where this interaction term was statistically significant, simple slope analyses were reported for good and poor sleepers. To control for the nested structure of our data (i.e., participants were nested within meeting points), we adjusted the standard errors to prevent them from being inflated due to clustering (multilevel analyses) [50]. All analyses were performed using Mplus. Statistical significance was set at *p* ≤ 0.05 [51].

## Results

**Fig. 1:**
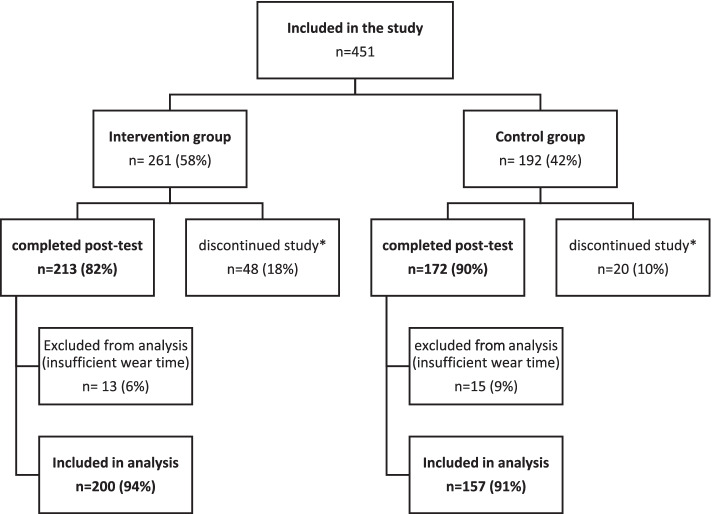
Flow chart of the controlled study. *discontinuation of the study due to sickness or personal reasons of the participants

In total, 451 adults 55+ years were enrolled in this study (58% intervention group and 42% control group). Post-test data were collected from 385 participants (55% intervention group and 45% control group). The data of 357 participants who met the determined minimum wear time eligibility criteria of the accelerometer measurement were included in the analyses (i.e., 93%). The intervention group consisted of 200 participants (56%) from 14 meeting points (mean age 71.3 years [SD ± 6.4]) and the control group consisted of 157 participants (44%) from 13 meeting points (mean age 72.2 years, [SD ± 6.7]). See Fig. 1 for a detailed overview of participants (flow chart).

Table 1 represents a detailed overview of the participants, the meeting points and the participants’ demographic characteristics. There were no statistically significant differences at baseline between the intervention and control groups in terms of demographic and health characteristics.

**Table 1:** Demographic and health characteristics at baseline of participants in the intervention and control groups

**Characteristic**		**Intervention group (*n* = 200 in 14 meeting points)**	**Control group (*n* = 157 in 13 meeting points)**	***P*-value**
**Age**	Mean, ±SD	71.30 ± 6.43	72.20 ± 6.70	.197
	(Range)	(55.72–89.53)	(56.18–94.12)	
**Sex, n(%)**	Male	55 (27%)	55 (35%)	.126
	Female	145 (72%)	102 (65%)	
**Education, n(%)**	Low	87 (44%)	82 (52%)	.207
	Medium	61 (30%)	48 (31%)	
	High	43 (21%)	24(15%)	
**Living situation, n(%)**	Living alone	40 (20%)	114 (27%)	.124
	Cohabitant	160 (80%)	156 (73%)	
**Professional status, n(%)**	Retired	187 (94%)	150 (96%)	.581
	Professionally active	10 (5%)	6 (4%)	
**Sleep status**	Good sleeper	99 (49%)	69 (44%)	.297
	Poor sleeper	101 (51%)	88 (56%)	
**Smoking, n(%)**	No	184 (92%)	151 (96%)	.309
	Yes	9 (5%)	4 (3%)	
**Alcohol, n(%)**	No	149 (75%)	115 (73%)	.468
	Yes	42 (21%)	39 (25%)	
**Caffeine before bedtime, n(%)**	No	171 (86%)	126 (80%)	.074
	Yes	21 (10%)	27 (17%)	
**Screen time before bedtime, n(%)**	No	11 (5%)	8 (5%)	.814
	Yes Missing	177 (89%)12 (6%)	144 (92%)0 (0%)	
**Sleep medication, n(%)**	No	172 (86%)	129 (82%)	.346
	Yes	25 (13%)	25 (16%)	
**Chronic condition, n(%)**	No	101 (51%)	87 (55%)	.296
	Yes	81 (40%)	55 (35%)	
	Missing	18 (9%)	15 (10%)	

T-test was used for the continuous variable (i.e., age) and Chi-square tests were used for the categorical variables (i.e., sex, education, living situation, professional status, sleep status); Only percentages of missing values > 5% are separately reported. Column percentages for the separate variables may therefore not add up to 100%. Significant *P*-values at α ≤ 0.05 are indicated with*.

Participants in the intervention group had significantly higher average baseline MVPA levels (28.5 min/day) compared with the control group (*p* < .001). There were no statistically significant differences between the intervention and control groups in terms of sleep characteristics at baseline (See Table 2). Although the total sleep time in both groups is lower than the average recommended total sleep time for older adults [52, 53], the average sleep efficiency in both groups is higher than the cut off value of 85%, which is considered to be efficient sleep [43, 54]. The average number of awakenings is comparable for both groups. The mean PSQI sleep quality score was 6 points out of 21 for all groups at all times, indicating a poor quality sleep [35, 43, 54]. Of the 357 participants included in the analyses, 168 were classified as good sleepers (47%) of which 69 participants in the control groups and 99 participants in the intervention group, and 189 were classified as poor sleepers (53%) %) of which 88 participants in the control groups and 101 participants in the intervention group.

**Table 2:** MVPA levels and sleep parameters at baseline (T0) and posttest (T1)

Outcomes	Parameter, mean (±SD)	Baseline (T0)			Posttest (T1)		
		Intervention (***n =*** 200)	Control (***n =*** 157)	*P*-value	Intervention (***n =*** 200)	Control (***n =*** 157)	*P*-value
**Physical activity**	MVPA (min/day)	158.42 (±92.45)	122.17 (±83.52)	< 0.001 *	179.75 (±83.75)	120.88 (±78.83)	< 0.001*
**Objective sleep measures**	Sleep efficiency (%)	93.90 (±2.80)	93.90 (±3.21)	.996	94.13 (±2.68)	94.38 (±2.81)	.401
	Total sleep time (min/night)	356.28 (±79.48)	344.42 (±80.90)	.166	359.93 (±76.62)	351.05 (±83.84)	.298
	WASO (min/night)	22.72 (±11.18)	21.53 (±12.10)	.336	21.3 (±10.51)	20.69 (±11.36)	.327
	Number of awakenings/night	9.95 (±4.70)	9.28 (±4.91)	.189	9.73 (±4.45)	8.90 (±4.70)	.089
**Subjective sleep measures**	Sleep quality, PSQI total score	6 (±4)	6 (±4)	.362	6 (±3)	6 (±4)	.677

Data are raw means. PSQI total score (range 0 to 21 points), score > 5 indicates poor-quality sleep [35, 43]. T0: measurement at baseline, T1: measurement at posttest; WASO: wake time after sleep onset; Significant *P*-values at α ≤ 0.05 are indicated with *.

### Effects of the lifestyle program ‘Lekker Actief’ on MVPA levels

There were significantly positive effects of ‘Lekker Actief’ on MVPA levels (see Table 3). Compared with the control group, MVPA levels of the intervention group increased with 38.44 min/day (CI 95% 12.55; 64.33, crude model), 36.49 min/day (CI 95% 9.38; 63.60, partially adjusted model) and; 43.02 min/day (CI 95% 12.83; 73.22, fully adjusted model). In all models, the interaction term program by sleeping status was nonsignificant.

**Table 3:** Effects of the lifestyle program ‘Lekker Actief’ on MVPA levels, compared with the control group

Physical activity	Model	B	(CI 95%)	p
**MVPA (min/day)**	crude model: overall	38.44	(12.55–64.33)	.004*
	partially adjusted model: overall	36.49	(9.38–63.60)	.008*
	fully adjusted model: overall	43.02	(12.83–73.22)	.005*

Crude model (condition and MVPA levels at T0 and T1), partially adjusted model (crude model adjusted for age, sex, education, living situation, professional status); fully adjusted model (partially adjusted model adjusted for smoking, use of alcohol, caffeine or screen time before bedtime, use of sleep medication, presence of chronic conditions). Significant *P*-values at α ≤ 0.05 are indicated with *; B = unstandardised beta.

### Effects of the lifestyle program ‘Lekker Actief’ on sleep

In general, there were no statistically significant main effects of the program on sleep outcomes (See Table 4). Only two interaction terms were significant. First, in the crude model predicting wake after sleep onset, the interaction term program by sleeping status was significant (B = − 3.43, CI 95% -6.78; − 0.08). Simple slope analysis revealed that among poor sleepers, the intervention program did not have any effect on wake after sleep onset (B = − 1.04, CI 95% -3.16; 1.09), neither was the effect of the intervention program on wake after sleep onset among good sleepers significant (B = 2.40, CI 95% -0.05; 4.84). Second, in the crude model predicting number of awakenings, the interaction term was also significant (B = − 1.80, CI 95% -3.40; − 0.20). Simple slope analysis revealed that among poor sleepers, the intervention program did not have any effect on the number of awakenings (B = − 0.36, CI 95% -1.29; 0.57), while the intervention increased the number of awakenings among good sleepers with 1.44 (CI 95% 0.49; 2.24). In all other models, the interaction terms were nonsignificant (all P’s > .06).

**Table 4:** Effects of the lifestyle program ‘Lekker Actief’ on objective and subjective sleep outcomes, compared with the control group

Sleep parameter	Model	B	(CI 95%)	p
**Sleep efficiency (%)**	crude model: overall	−0.25	(− 0.70; 0.21)	.293
	partially adjusted model: overall	−0.27	(−0.78; 0.24)	.304
	fully adjusted model: overall	−0.12	(−0.60; 0.37)	.641
**Total sleep time (minutes/night)**	crude model: overall	2.16	(−17.74; 22.05)	.832
	partially adjusted model: overall	1.82	(−16.24; 19.88)	.843
	fully adjusted model: overall	4.99	(−11.85; 21.83)	.561
**Wake after sleep onset (minutes/night)**	crude model: overall	0.48	(−1.15; 2.11)	.563
	partially adjusted model: overall	0.64	(−1.20; 2.47)	.498
	fully adjusted model: overall	− 0.14	(−1.97. 1.69)	.880
**Number of awakenings/night**	crude model: overall	0.42	(−0.12; 0.97)	.129
	partially adjusted model: overall	0.56	(−0.06; 1.17)	.076
	fully adjusted model: overall	0.26	(−0.36; 0.89)	.410
**PSQI sleep quality (total score)**	crude model: overall	0.07	(−0.39; 0.52)	.772
	partially adjusted model: overall	0.03	(−0.42; 0.48)	.906
	fully adjusted model: overall	0.04	(−0.57; 0.65)	.897

Crude model (condition and MVPA levels at T0 and T1), partially adjusted model (crude model adjusted for age, sex, education, living situation, professional status); fully adjusted model (partially adjusted model adjusted for smoking, use of alcohol, caffeine or screen time before bedtime, use of sleep medication, presence of chronic conditions). Significant *P*-values at α ≤ 0.05 are indicated with *; B = unstandardised beta.

## Discussion

The aims of this controlled pretest-posttest study were twofold: 1) examine the effects of a lifestyle program on device-measured moderate-to-vigorous physical activity and objective and subjective sleep (i.e., objective sleep outcomes: sleep efficiency, total sleep time, WASO and number of awakenings and subjective sleep quality (PSQI)) in adults aged 55+ years; and 2) examine if the effects differed between good and poor sleepers. In what follows, we will discuss possible reasons for the fact that although the program successfully increased MVPA levels, only few significant and clinically relevant findings were observed for sleep.

### Effects of ‘Lekker Actief’ on MVPA and sleep

The program was effective in increasing MVPA levels with 43 min per day in the fully adjusted models. This is not only statistically significant, but also a relevant increase. Recent PA guidelines for older adults indicate that a minimum level of 150 min MVPA per week is needed for health benefits [29, 30]. If we translate this to a daily level to put the effects of ‘Lekker Actief’ into perspective, this means that the recommendation of 21 min of MVPA (150 min/7 days) is almost achieved twice after participation in this program. Despite these significant increased MVPA levels, there were no significant effects of ‘Lekker Actief’ on sleep. Thus, even though more controlled studies, as described in a recent review, previously established positive effects of PA on WASO, sleep quality, sleep latency and sleep disturbances [5], these findings could not be confirmed in our intervention study in a real-life setting.

Given the growing life expectancy and the increased prevalence of sleep problems at higher age, it is crucial to detect which programs could benefit sleep for **good and poor sleepers** separately. Only two interactions between program and sleep condition were significant: one when predicting wake after sleep onset and one when predicting the number of awakenings. Yet, these interactions were only significant in the crude models and not in the partially or fully adjusted model. Furthermore, the simple slopes where either not significant (regarding wake after sleep onset), or clinically not relevant. In general, we can therefore conclude that the intervention effect did not differ between good and poor sleepers. Interestingly, in contrast to our study, previous research reported a positive effect of regular exercise on sleep in older adults that were considered **poor sleepers** [27, 28]. More specifically, a study by Yang et al. (2012) reviewed studies that examined the benefits of exercise training programs for sleep in middle aged and older adults with sleep problems measured by PSQI and polysomnography [27]. These authors concluded that sleep latency, sleep medication and subjective sleep quality were improved after following a moderate to high intensity exercise training program. A more recent review by Lowe et al. (2019) examined the effects of daily exercise sessions on sleep in adults with insomnia [28]. This review showed that after following an exercise intervention, subjective sleep quality, sleep latency and sleep efficiency was improved, regardless of the exercise intensity. Even though these reviews focused on exercise programs, and not on MVPA as was the case in our study, our finding that ‘Lekker Actief’ did not affect sleep outcomes is in contrast with the conclusion by Lowe et al. (2019). Potential reasons for the discrepancy in findings between those studies and the present study will be discussed below.

With respect to good sleepers, Vanderlinden et al. (2020) reviewed the effects of PA programs on sleep in older adults without sleep problems and concluded that quality of sleep, sleep latency, sleep disturbances, WASO, sleep duration and sleep efficiency were improved after following a PA program in more controlled settings [5]. Our present intervention study did not confirm these previously found positive effects for these sleep outcomes.

### Possible reasons for the few significant and clinically limited findings in terms of sleep

This study found only few statistically significant and clinically relevant results in terms of sleep. Three possible reasons that we will discuss below include: the real-life context of the ‘Lekker Actief’ program, the use of accelerometry to measure MVPA and sleep and the 24 h continuum.

#### The real-life context of the ‘Lekker Actief’ program

The real-life context of this program represents a natural setting, which might have caused a dilution of the effects on sleep outcomes that were found in more controlled studies. This explanation is in line with the conclusions of a previously published review on the translation of program effectiveness to real-life programs in diabetes type 2 patients [55]. The authors of this review identified three key factors that might explain the difference between results from more controlled, lab-based studies and real-life studies: (1) participation levels in the program, (2) intensity and frequency of the program (3); implementation fidelity.

First, in terms of the participation levels in our study, it was not possible for us to register the session attendance of participants because of two reasons. First, OKRA SPORT+, who organized the program, wanted to keep the participation burden for the local organizers as low as possible. Second, given the self-determination theory, which formed the theoretical backbone of this program, controlling for sessions attendance might have a negative effect on the self-perceived autonomy of the participants. Consequently, we were not able to control for program adherence in our analyses. It may have been the case that there would be an effect on sleep in people with higher participation rates, as has previously been shown in PA intervention studies [56, 57]. Second, with regard to the intensity, frequency and type of exercise, the ‘Lekker Actief’ program was developed and organized by the socio-cultural organization (OKRA SPORT+). Therefore, we did not have a possibility to change or control the content of the program itself. The different components in this real-life lifestyle program (walking, muscle strengthening and healthy nutrition) were not offered in each meeting point in the same standardised way or dosage which could have impacted the effects on MVPA or sleep. In addition, the 12-week duration of the program might have been too short to establish more and larger effects on sleep, considering that PA programs with a duration of six months and more have shown larger effects on sleep outcomes [5]. In addition to this second key factor, the attained frequency (session attendance) and intensity (compliance with MVPA) of the program was not monitored by use of exertion scales or heart rate measurement during the program. Monitoring intensity may have provided participants with feedback and the possibility to adjust their intensity levels if they did low intensity rather than MVPA. We cannot exclude the possibility that the dose of MVPA might not have been high enough to replicate the positive effects on sleep observed in more strictly controlled studies. Furthermore, ‘Lekker Actief’ was intended to increase MVPA levels and this was communicated to the participants. The lack of significant positive effects on sleep could also be based upon the fact that our participants were not focused on improving sleep during the program, as was the case in other studies that did show significant and positive effects on sleep [5].

Third, in terms of implementation fidelity, no quality assurance was implemented in our study to assess the extent to which the program was delivered in real-life as originally intended by the organization OKRA SPORT+. In order to successfully facilitate translation of established effects from more controlled lab-based studies in to community settings, Miller et al. (2012) emphasized that program fidelity, staff and organizational capacity and engagement as well as a program evaluation constitute important steps in this translation process [58]. Future organisers of real-life lifestyle programs are encouraged to focus on these criteria.

#### The use of accelerometry to measure MVPA and sleep

Another potential reason for the clinically limited findings is the use of accelerometry, more specifically Actigraphs, to objectively measure sleep. Although these devices are shown to be reliable in measuring sleep in this population group, polysomnography could have provided more in-depth data in terms of sleep stages and might also have been more sensitive to detect smaller changes in sleep compared with the Actigraph. These actigraphs are less expensive and easier to use when compared with polysomnography, which makes this method more realistic to use when assessing sleep in larger samples [37]. Furthermore, polysomnography is performed in a clinical or controlled sleep lab setting, which does not represent a natural sleep environment compared with accelerometry measurement, which can be performed in the participants’ own bed (room). Finally, the placement of the accelerometer (i.e. wrist-worn) could have affected the effects in this study [59]. It is known that hip-worn accelerometers reproduce much smaller acceleration values during walking than wrist-worn accelerometers which in turn could result in a misclassification of LPA as MVPA. Indeed, a recent study by Bammann et al. (2021) in which older participants wore Actigraph GT3x + accelerometers on the ankle, hip and wrist, concluded that there was a higher inter-individual variability of arm movement (wrist-worn accelerometer) compared with core body (hip or ankle-worn accelerometer) during activity [40]. In this study, we used this wrist-worn placement for measures of both MVPA levels and sleep. In order to identify MVPA levels and avoid misclassification of LPA as MVPA, we made use of a tri-axial vector magnitude (VM3) cut-point (≥3268 CPM) for wrist-worn actigraphy in older adults [40].

Although wrist-worn accelerometry is preferred above waist-worn accelerometry when assessing sleep [60, 61], waist-worn accelerometry is preferred for measuring PA [62, 63]. Previous studies also showed valid results when using wrist-worn accelerometers for measuring physical activity [21, 36, 41].

#### Time and type of PA and the circadian rhythm

Previous studies in more controlled settings have already established positive effects of PA programs on sleep in older adults. However, as already discussed in the review by Vanderlinden et al. (2020), it remains unclear how timing and type of exercise might impact sleep [5].

Although previous studies in more controlled settings have already established positive effects of PA programs on sleep in older adults, it remains unclear how timing and type of exercise might impact sleep [5]. Exercising at different times throughout the circadian rhythm could result in different effects on sleep. With regard to time of PA, previous research has already shown that exposure to bright daylight improves sleep [19, 20]. Furthermore, vigorous intensity physical activity prior to bedtime could worsen sleep outcomes as it would increase bodily temperature, which in turn is not conducive for sleep [64, 65]. Regarding the type of PA, different types of PA (i.e. aerobic exercise versus resistance training) might go along with different effects on sleep, although evidence on the ideal type of exercise for sleep in older adults is still lacking [66]. Finally, individual characteristics of participants (i.e. circadian rhythm, chronotypes) could also have impacted the effects of the program on sleep [19, 20, 67]. Although these factors may have affected the effects of PA on sleep, we did not control for participants that performed other types of exercise on top of the components of this real-life program, nor did we control for the exact time of the day when participants were physically active, or collected data about their actual chronotype.

### Strengths and limitations

This study has several strengths and limitations. Strengths include the focus on a lifestyle program in a real-life setting, which favours the ecological validity of the findings; an intervention in a large sample size of older adults and; the device-based measurement of MVPA and the availability of subjective and objective sleep data. There are also several limitations. First, this study was a non-randomised controlled trial, which may have caused bias. Despite this lack of randomisation, we did match participants of the intervention group with the control groups in terms of regions and the number of participants per meeting points. Second, we are not sure that the dose of PA in this program was delivered as initially intended, given that we were not able to register and monitor the session attendance and compliance with intensity of physical activity during the program. Consequently, we could not control for these indicators in the analysis in this study, which might have caused an underestimation of the effects and associations. Third, although we used several objective and subjective sleep outcomes, we performed several analyses in different models. Therefore, the possibility of a type 1 error cannot be excluded. Based upon a set α 0.05, there is a 5% possibility that significant findings might be based on coincidence rather than on significance. Finally, the use of wrist-worn devices is a limitation as this wear-location might lead to an overestimation MVPA time, which is reflected in high MVPA levels in both conditions at baseline [40, 59].

### Generalizability and implications

As the lifestyle program ‘Lekker Actief’ was not intended to improve sleep, its components (i.e., walking program, muscle strengthening program and healthy nutrition) were mainly focused on increasing MVPA levels and overall health. Based on findings from more controlled studies examining the effects of PA and exercise programs on sleep, one of the aims of this study was to explore whether real-life lifestyle programs that offer MVPA could also be used to promote sleep in older adults. Only participating meeting points in the real-life lifestyle program ‘Lekker Actief’ were eligible for the inclusion in the intervention group. This selective sampling strategy could have caused selection bias. Finally, when comparing baseline measures of MVPA levels and sleep between conditions and with the general population, it became clear that the older adults in this study were generally more active when compared with the Belgian population of the same age [7, 68]. Moreover, the older adults in the intervention group were significantly more active at baseline when compared with the control group. Therefore, the generalizability of our data to more inactive older adults can be questioned. It could be possible that in older adults with lower MVPA baseline levels, increases in MVPA could result in improved sleep as has been observed in previous studies.

It is clear from this study that the findings from previous more controlled studies do not equally translate to real-life studies like ‘Lekker Actief’ with regard to positive effects on sleep. In order to facilitate this translation to a real-life setting, future lifestyle programs for older adults should include strategies to monitor session attendance and compliance with the intended program intensity of each program session to ensure that the intended dose of MVPA is achieved. Although there are indications for a different effect of physical activity on good and poor sleepers, this was not confirmed in the present study. It is recommended that, in order to increase focus on improving sleep in these lifestyle programs, a sleep enhancing component (i.e. sleep hygiene advice, cognitive behavioural therapy or relaxation), preferably provided by a licensed sleep therapist, might increase the overall impact on sleep [69–72].

## Conclusion

This intervention study showed that ‘Lekker Actief’, a lifestyle program in a real-life setting for adults aged 55+ years, was effective in increasing MVPA levels among older adults, but it showed no positive effect on sleep outcomes. Effects did not differ between good or poor sleepers. Future lifestyle programs that aim to improve sleep in older adults by means of increasing MVPA levels, are advised to ensure that participants receive a sufficient dose of physical activity to positively affect sleep, potentially in combination with a sleep enhancing component.

## Data Availability

The dataset used and analysed in this article is available from the corresponding author on reasonable request.

## Abbreviations

CPM: counts per minute
CSEP: Canadian Society for exercise Physiology
ISCED: International Standard Classification of Education
LPA: Light intensity physical activity
Min: Minutes
MPA: Moderate intensity physical activity
MVPA: Moderate to vigorous intensity physical activity
PA: Physical activity
PSQI: Pittsburgh Sleep Quality Index
VPA: Vigorous intensity physical activity
VM3: Tri-axial vector magnitude
WASO: Wake time after sleep onset
WHO: World Health Organization
